# Controlling cholera in the Ouest Department of Haiti using oral vaccines

**DOI:** 10.1371/journal.pntd.0005482

**Published:** 2017-04-14

**Authors:** Alexander Kirpich, Thomas A. Weppelmann, Yang Yang, John Glenn Morris, Ira M. Longini

**Affiliations:** 1 Department of Molecular Genetics and Microbiology, College of Medicine, University of Florida, Gainesville, Florida, United States of America; 2 Emerging Pathogens Institute, University of Florida, Gainesville, Florida, United States of America; 3 Herbert Wertheim College of Medicine, Florida International University, Miami, Florida, United States of America; 4 Department of Biostatistics, College of Public Health and Health Professions and College of Medicine, University of Florida, Gainesville, Florida, United States of America; 5 Department of Medicine, College of Medicine, University of Florida, Gainesville, Florida, United States of America; Sanofi Pasteur, FRANCE

## Abstract

Following the 2010 cholera outbreak in Haiti, a plan was initiated to provide massive improvements to the sanitation and drinking water infrastructure in order to eliminate cholera from the island of Hispaniola by 2023. Six years and a half billion dollars later, there is little evidence that any substantial improvements have been implemented; with increasing evidence that cholera has become endemic. Thus, it is time to explore strategies to control cholera in Haiti using oral cholera vaccines (OCVs). The potential effects of mass administration of OCVs on cholera transmission were assessed using dynamic compartment models fit to cholera incidence data from the Ouest Department of Haiti. The results indicated that interventions using an OCV that was 60% effective could have eliminated cholera transmission by August 2012 if started five weeks after the initial outbreak. A range of analyses on the ability of OCV interventions started January 1, 2017 to eliminate cholera transmission by 2023 were performed by considering different combinations of vaccine efficacies, vaccine administration rates, and durations of protective immunity. With an average of 50 weeks for the waiting time to vaccination and an average duration of three years for the vaccine-induced immunity, all campaigns that used an OCV with a vaccine efficacy of at least 60% successfully eliminated cholera transmission by 2023. The results of this study suggest that even with a relatively wide range of vaccine efficacies, administration rates, and durations of protective immunity, future epidemics could be controlled at a relatively low cost using mass administration of OCVs in Haiti.

## Author summary

After the introduction of toxigenic *Vibrio cholerae* O1 to the Caribbean nation of Haiti in 2010, the country experienced the largest national cholera outbreak in recent history. With the help of the international community, over $500,000,000 USD was pledged to finance massive improvements in drinking water and sanitation infrastructure with the goal of eliminating cholera transmission by 2023. Over six years later, cholera transmission continues, with little evidence that any such improvements have been made. In this study, the use of oral cholera vaccines (OCVs) to reduce the spread of the initial outbreak and to eliminate future transmission was investigated using a novel mathematical modeling framework. Critical parameters including vaccine efficacy, vaccine administration rates, and duration of protective immunity were evaluated to provide guidelines for the administration of a National Cholera Vaccine Program in Haiti. Provided the vaccines are at least 60% effective, all simulations of mass vaccination programs successfully eliminated cholera by 2023, most within a time frame of only a few years. Not only do the findings support the use of OCVs to eradicate transmission of cholera in Haiti, they indicate that OCVs should be mobilized to quash future outbreaks in other countries using active vaccine administration campaigns.

## Introduction

Since the introduction of toxigenic *Vibrio cholerae* O1 to Haiti in October 2010, an altered El Tor biotype has spread throughout the country causing over 750,000 cases and resulted in the largest national cholera epidemic in recent history [1] [2] [3]. Six years later, cholera transmission continues, albeit at a diminished rate; with mounting evidence that environmental reservoirs of *V. cholerae* have been established in the warm, tropical surface waters of Haiti [4] [5]. The relatively high proportion (greater than 40%) of the population without access to improved drinking water sources [6], increased isolation frequency of toxigenic *V. cholerae* O1 in the environment [7], and the waning of immunity acquired by those previously infected with El Tor cholera [8] provide the necessary conditions for seasonal outbreaks in the near future.

In the absence of a complete overhaul of the nation’s drinking water and sanitation infrastructure, it has been suggested that mass vaccinations by oral inactivated whole-cell cholera vaccine (OCV) could have completely prevented the ongoing epidemic and be used to mitigate future transmission of cholera in Haiti [9] [10]. OCVs have demonstrated considerable protective efficacy (approximately 65%) in rigorously designed clinical trials conducted in India [11] [12], Bangladesh [13] [14], and Vietnam [15] [16]. Despite their efficacy, any large scale vaccination campaign at the beginning of the Haitian outbreak was not feasible due to the limited supply of OCVs [17], which at that time were not pre-qualified by the World Health Organization (WHO) for the control of cholera outbreaks. Though the WHO acknowledged the availability of two OCVs that had documented efficacy, feasibility, and cultural acceptance; they concluded that the relatively short duration of protection from OCVs (roughly 2–5 years), should not disrupt other health interventions with long-term effects, such as providing appropriate medical treatment, implementing interventions to improve drinking water and sanitation, and mobilizing communities to adopt more hygienic practices [18]. Currently, the position of the WHO is that vaccination should be an effective component of control strategies in cholera-endemic countries, which they define as countries where culture-confirmed cholera has been detected in three of the past five years with an incidence of at least 1/1000 population members in any of those years; both of which now apply to Haiti [19].

With ongoing transmission of cholera six years after the initial outbreak and epidemiological characteristic that resemble an endemic rather than epidemic state, the use of OCVs to control cholera in Haiti warrants further investigation. In the current study, we have used mathematical models to accomplish three objectives:(i) determine the effect that a reactive vaccination campaign would have had on the initial outbreak; (ii) use historical meteorological measurements and a recently developed data-driven model to simulate future cholera outbreaks; (iii) explore the potential benefits of mass immunization programs on simulated future outbreaks of cholera. These findings may assist in planning future strategic vaccination programs by providing estimates of critical operational parameters necessary to finally control cholera transmission in Haiti.

## Materials and methods

**Fig 1:**
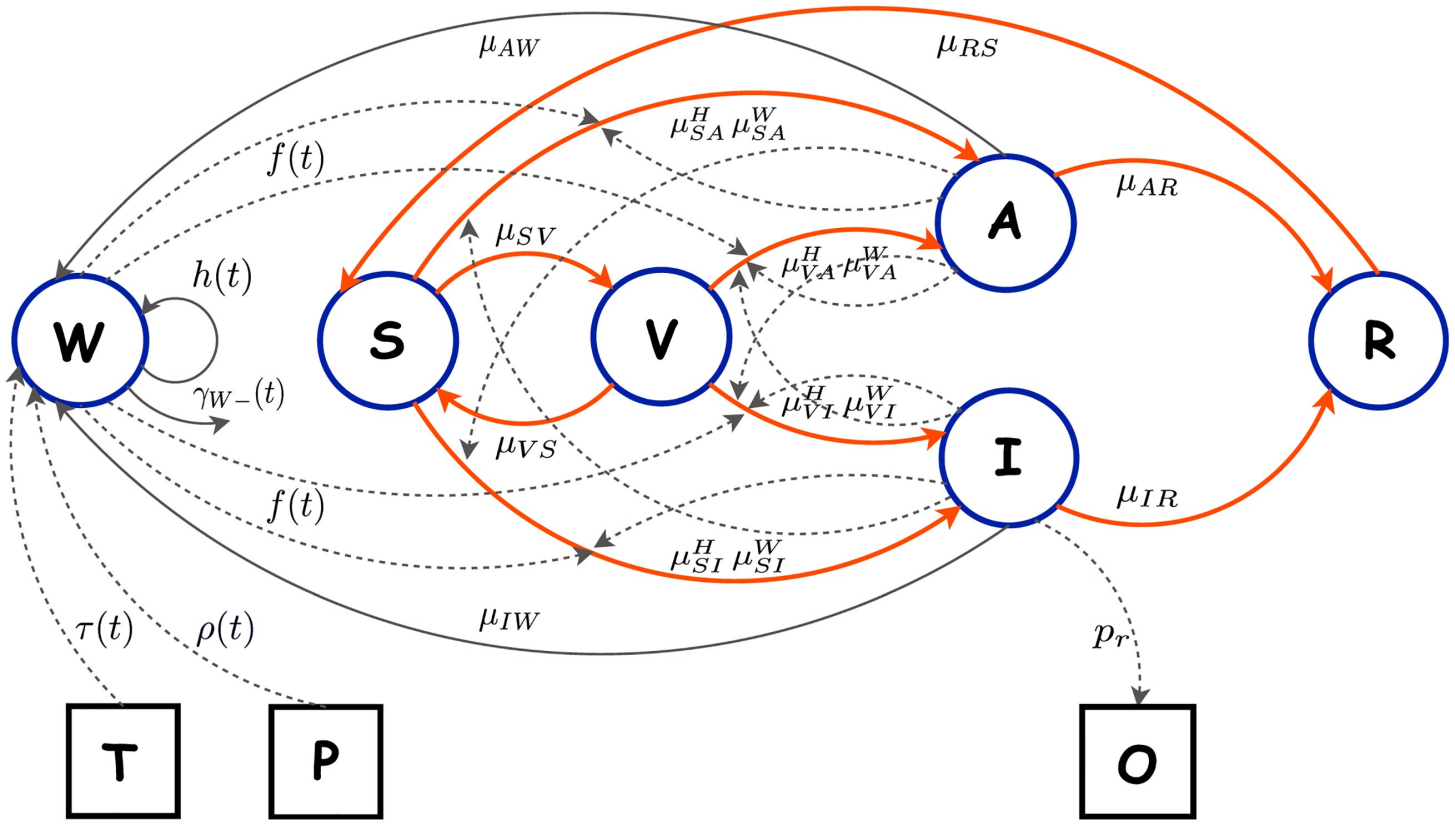
The complete diagram of the compartment model with vaccination intervention is presented. The unobserved human compartments and environmental compartment *W* of the *SIRS* model are denoted by circles while the observed data are represented by square boxes. Humans move through the unobserved *SIRS* compartments and interact with the unobserved environmental reservoir *W* which is influenced by the ambient temperature *T* and precipitation *P*. The unobserved number of asymptomatic *A* and symptomatic *I* cholera infections are linked to the observed incidence *O*. Solid lines denote either the movement of humans between compartments (thick orange lines) or the bacteria shedding and multiplication in the environment (thin grey lines). Dashed lines represent the relationships between different elements of the dynamic model that are incorporated via the system of model equations. Susceptible individuals *S* move to the vaccinated compartment *V* for the duration of immunity provided by the vaccine; where vaccinated individuals can become infected based on the efficacy of the vaccine.

A dynamic compartment model was used to simulate the impact of multiple vaccine intervention campaigns on cholera transmission in the Ouest Department of Haiti. In this manuscript, ‘elimination of transmission’ and ‘control of cholera’ are used synonymously to mean zero new human cases, which is not equivalent to the elimination of cholera completely, i.e., the absence of the causative bacterium from both humans and the environment. This model, referred to as the intervention model, is an extention of a previously developed framework by incorporating a vaccinated human compartment to investigate various vaccination interventions that investigate parameters crucial for successful control of cholera using OCVs [20]. In addition, to make long term epidemic predictions, birth and death rates were included in the current model to reflect natural population dynamics. The intervention model facilitates the movement of humans between the susceptible and infected compartments via both short- and long-cycle transmission. The human-to-human transmission route (short-cycle) represents the direct contact between humans and the environment-to-human (long-cycle) route represents transmission via consumption of surface water that serves as an environmental reservoir of toxigenic *V. cholerae* O1. The concentration of bacteria in the environmental compartment is influenced by the influx of toxigenic *V. cholerae* O1 from the feces of infected humans, bacterial proliferation and survival in response to environmental factors, and bacterial death. After exposure, infected humans move into either asymptomatic or symptomatic compartments that correspond to different levels of infectivity and course of illness. The intervention model diagram is presented in Fig 1.

The following notations for the compartments were used:

- *S*(*t*)—the size of the susceptible compartment at a given time *t*.
- *A*(*t*)—the size of the asymptomatic compartment at a given time *t*.
- *I*(*t*)—the size of the symptomatic compartment at a given time *t*.
- *R*(*t*)—the size of the recovered compartment at a given time *t*.
- *V*(*t*)—the size of the vaccinated compartment at a given time *t*.
- *W*(*t*)—bacteria concentration in water at a given time *t* (environmental compartment).

In this model, the rate of change in the bacterial concentration of the environmental compartment is affected by three main processes: an influx of toxigenic *V. cholerae* O1 from the feces of infected humans (influenced by precipitation), multiplication of the pathogen in the environment (influenced by temperature and precipitation), and bacterial death. These processes are modeled by functions *g*(*t*), *h*(*t*), and a time-varying bacterial death rate *γ*_*W*−_(*t*). The functions *g*(*t*) and *h*(*t*) have the form:

$$\begin{array}{c} \begin{array}{cc} & g\left(t\right)=\frac{\rho (t)}{\delta +\rho (t)},\\ & h\left(t\right)=\alpha \exp\left[-\frac{{\left(\rho \left(t\right)-\rho_{c}\right)}^{2}}{2\sigma^{2}}\right]+\beta \tau \left(t\right), \end{array} \end{array}$$ (1)

where *τ*(*t*) is the average ambient temperature and *ρ*(*t*) is the total precipitation during week *t*. Other notations used in Eq (1) are the threshold parameter *δ* and calibration parameters *α*, *ρ*_*c*_, *σ*, and *β*.

To reflect the effect of the of the environmental concentration of the bacteria on human infection we define a function:

$$\begin{array}{c} f\left(t\right)=\frac{W(t)}{\kappa +W(t)}, \end{array}$$ (2)

where *κ* is the threshold parameter and *χ* is the cap of the concentration of bacteria in the environmental compartment *W*. Another function *m*(*t*) is used together with *W*(*t*) to regulate excessive bacterial growth:

$$\begin{array}{c} \begin{array}{cc} & m\left(t\right)=1-\frac{W(t)}{\chi }=\frac{\chi -W(t)}{\chi }. \end{array} \end{array}$$ (3)

The deterministic model is stated in terms of the ordinary differential equations (ODE). In the model statement transition rates have notations *μ* and *γ*, with corresponding subscripts indicating the involved compartments of the model and the direction of the flow between them. Superscripts indicate the nature of the flow between compartments where *H* represents human-to-human transmission and *W* represents environment-to-human transmission. The birth and death rates of human hosts are denoted by *μ*_*H*+_ and *μ*_*H*−_. The vaccination rate is controlled by parameter *μ*_*SV*_, duration of immunity by *μ*_*VS*_, and the vaccine efficacy by parameters $\mu_{VI}^{H}$, $\mu_{VI}^{W}$, $\mu_{VA}^{H}$ and $\mu_{VA}^{W}$. The total population size is denoted as *N*. The intervention model equations have the form:

$$\begin{array}{c} \begin{array}{cc} & \frac{dS(t)}{dt}=\mu_{H+}N+\mu_{RS}R\left(t\right)+\mu_{VS}V\left(t\right)-\left(\mu_{SA}^{W}+\mu_{SI}^{W}\right)S\left(t\right)\frac{W(t)}{\kappa +W(t)}\\ & -\left(\mu_{SA}^{H}+\mu_{SI}^{H}\right)S\left(t\right)\left(A\left(t\right)+I\left(t\right)\right)-\left(\mu_{SV}+\mu_{H-}\right)S\left(t\right)\\ & \frac{dV(t)}{dt}=\mu_{SV}S\left(t\right)-\left(\mu_{VA}^{W}+\mu_{VI}^{W}\right)V\left(t\right)\frac{W(t)}{\kappa +W(t)}\\ & -\left(\mu_{VA}^{H}+\mu_{VI}^{H}\right)V\left(t\right)\left(A\left(t\right)+I\left(t\right)\right)-\left(\mu_{VS}+\mu_{H-}\right)V\left(t\right)\\ & \frac{dA(t)}{dt}=\left[\mu_{SA}^{W}S\left(t\right)+\mu_{VA}^{W}V\left(t\right)\right]\frac{W(t)}{\kappa +W(t)}\\ & +\left[\mu_{SA}^{H}S\left(t\right)+\mu_{VA}^{H}V\left(t\right)\right]\left(A\left(t\right)+I\left(t\right)\right)-\left(\mu_{AR}+\mu_{H-}\right)A\left(t\right)\\ & \frac{dI(t)}{dt}=\left[\mu_{SI}^{W}S\left(t\right)+\mu_{VI}^{W}V\left(t\right)\right]\frac{W(t)}{\kappa +W(t)}\\ & +\left[\mu_{SI}^{H}S\left(t\right)+\mu_{VI}^{H}V\left(t\right)\right]\left(A\left(t\right)+I\left(t\right)\right)-\left(\mu_{IR}+\mu_{H-}\right)I\left(t\right)\\ & \frac{dR(t)}{dt}=\mu_{AR}A\left(t\right)+\mu_{IR}I\left(t\right)-\left(\mu_{RS}+\mu_{H-}\right)R\left(t\right)\\ & \frac{dW(t)}{dt}=g\left(t\right)\left(\mu_{AW}A\left(t\right)+\mu_{IW}I\left(t\right)\right)+h\left(t\right)m\left(t\right)W\left(t\right)-\gamma_{W-}\left(t\right)W\left(t\right) \end{array} \end{array}$$ (4)

To avoid identifiability issues, additional assumptions were made regarding the parameters in model (4). Selected parameters were assigned fixed values based on biological processes obtained via literature review and estimation from the previous study (please refer to the S1 Text). Symptomatic and asymptomatic transmission and recovery rates were linked to each other in a fixed deterministic manner; with $\mu_{SA}^{W}=3\mu_{SI}^{W}$, $\mu_{SA}^{H}=3\mu_{SI}^{H}$ and *μ*_*AR*_ = *μ*_*IR*_. To ensure model equilibrium, death and birth rates were assumed to be the same *μ*_*H*+_ = *μ*_*H*−_ and equal to the annual population birth rate of 22.83 births per 1,000 people [21].

Different levels of vaccine efficacy (VE) were considered [22]. In model (4) vaccine efficacy is controlled by the parameter *ϑ* = 1 − *VE*. For example, *ϑ* = 0.4 corresponds to a vaccine efficacy of 0.6 or 60% if measured in percentages. In the intervention model, the infection rates for vaccinated individuals are controlled by parameter *ϑ*, which has the form $\mu_{VA}^{H}=3\mu_{VI}^{H}=3\vartheta \mu_{SI}^{H}$ and $\mu_{VA}^{W}=3\mu_{VI}^{W}=3\vartheta \mu_{SI}^{W}$. Future epidemics in the absence of vaccination were predicted with *VE* = 0 (*ϑ* = 1).

The weekly reported incidence for the Ouest Department of Haiti (including Port-au-Prince) from October 17, 2010 until April 27, 2014 was used to estimate the effects of temperature and precipitation on environmental concentrations of toxigenic *V. cholerae* O1 and cholera incidence as described previously [20]. The challenges in the computation of the basic reproductive number $R_{0}$ and the effective reproductive number R(t) have been discussed in detail in previous work [20]. Incidence data were collected by Ministry of Health Haiti (Ministère de la Santé Publique et de la Population (MSPP) [23] and compiled by the Pan American Health Organization (PAHO) [24]. Reported incidence data were adjusted for underreporting with the assumption that only 75 percent of cases were reported. Daily records of precipitation (in millimeters) were collected from the Tropical Rainfall Measuring Mission (TRMM) [25] along with daily temperature readings (in Celsius) collected by the Port-au-Prince airport monitoring station (IATA: PAP). Environmental data for future dates were extrapolated from the daily average of the observed readings between October 11, 2010 and June 21, 2014 (Fig A and B in S1 Text). Please refer to the S1 Text for more details about the extrapolation of future environmental data.

Simulations for estimation and prediction were performed based on the Markov Chain assumptions using the set of Eq (4) as infinitesimal generators and the Gillespie algorithm [26]. Three main simulation scenarios were considered for the study: (1) No intervention was implemented; (2) A reactive vaccination campaign was implemented five weeks after the initial 2010 outbreak; (3) An OCV intervention campaign was started on January 1, 2017. Since the third scenario was the most relevant from public health and policy prospective, extensive sensitivity analyses with different vaccination strategies and vaccine efficacy parameters were performed based on this scenario. The population size used for simulation was three million, and the total number of simulations for each epidemic scenario considered was 500. The starting points for each epidemic were generated from uniform distributions with the distribution means corresponding to the number of reported cases during the first week of the observed epidemic. To cover a reasonable range of vaccination pace and sustainability of vaccine protection, we considered six possible values of *μ*_*SV*_ to reach expected waiting times to vaccination of 5, 10, 20, 30, 40 and 50 weeks and three possible values of *μ*_*V S*_ corresponding to an average of two, three and five years of protection after vaccination. The exact distributions for waiting time to vaccination and the duration of protection are given in Tables C and D in the S1 Text. Details about all the simulation parameter settings and the results of sensitivity analyses are provided in S1 Text.

## Results

### Effects of reactive vaccination campaigns during the initial outbreak

**Fig 2:**
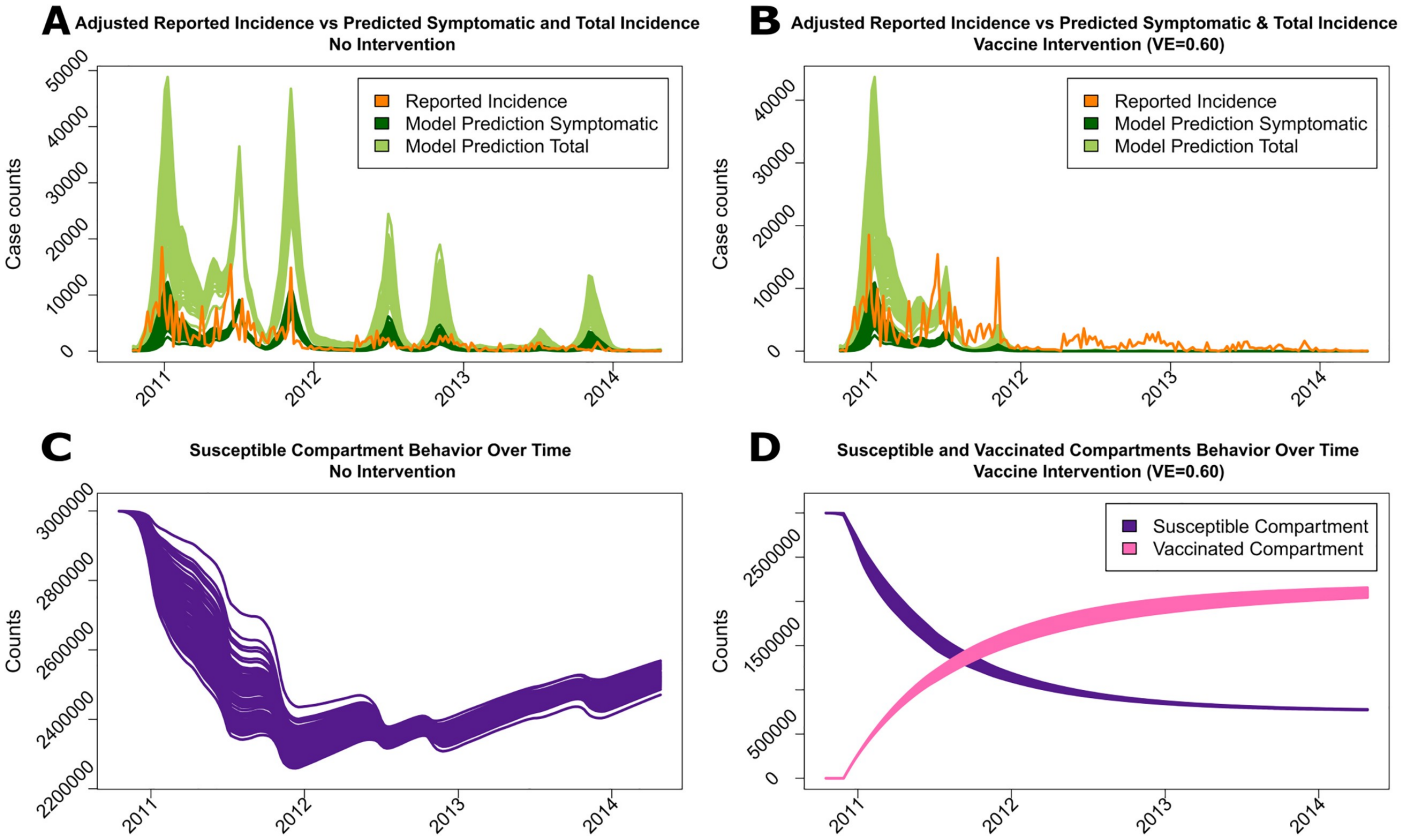
The upper panels show the observed incidence of symptomatic cholera cases adjusted for under-reporting (orange), the model-fitted incidence of symptomatic cases (dark green), and both symptomatic and asymptomatic infections (light green) from October 2010 to April 2014, without (A) and with (B) vaccine intervention initiated on November 17, 2010 (five weeks after the onset of the epidemic). The numbers of susceptible (purple) and vaccinated (pink) individuals are shown in the lower panels, without (C) and with (D) vaccine intervention. The average waiting time for a susceptible individual to be vaccinated is 50 weeks, the vaccine efficacy is 60%, and the average duration of protective immunity is three years.

The epidemics produced with and without mass vaccination interventions are displayed in Fig 2. The adjusted reported incidence displayed in orange was compared with the model-produced symptomatic incidence displayed in dark green. The adjusted reported incidence was well captured by the the model-produced symptomatic incidence, with a slight overestimation of the number of reported cases after the second year of the epidemic (Fig 2A). As shown, the total (symptomatic and asymptomatic) number of infections (light green) estimated by the model illustrates that the underlying cholera epidemic in Haiti was actually much larger than reported. All three million people in the Ouest Department were assumed to be susceptible to cholera at the onset of the 2010 outbreak. The number of susceptible individuals reached its lowest value, around 75%, by the end of 2011 and then started to grow gradually to about 80% by the middle of 2014 (Fig 2C). With the addition of the vaccinated compartment to the estimation model, the effect of a reactive vaccination campaign initiated five weeks after the beginning of the outbreak using model (4) was investigated. In this campaign, the average time required for a susceptible individual to be vaccinated (referred to hereafter as ‘waiting time’) was 50 weeks, the vaccine efficacy was 60%, and the duration of protective immunity lasted an average of three years. The exact time periods required to reach certain levels of vaccine coverage in the population for an average of 50-week waiting time are displayed in the first row of Table C in the S1 Text. The simulations suggest that the epidemic could have been completely controlled by August 5, 2012 (95% CI: March 11, 2012; December 16, 2012) had vaccination started after the fifth week of the outbreak (Fig 2B). Even though it takes some time to reach complete control, the reduction in the number of new cholera cases becomes visible within a few weeks after the initiation of the vaccination campaign (Fig 2B). This effect can be attributed to herd immunity in this population, where individuals who have not been vaccinated have a lower risk of infection due to the reduction in the number of human hosts available to spread the causative bacterium and a decrease in the environmental concentrations of toxigenic *V. cholerae* O1.

### Construction of future cholera epidemics for testing OCV interventions

**Fig 3:**
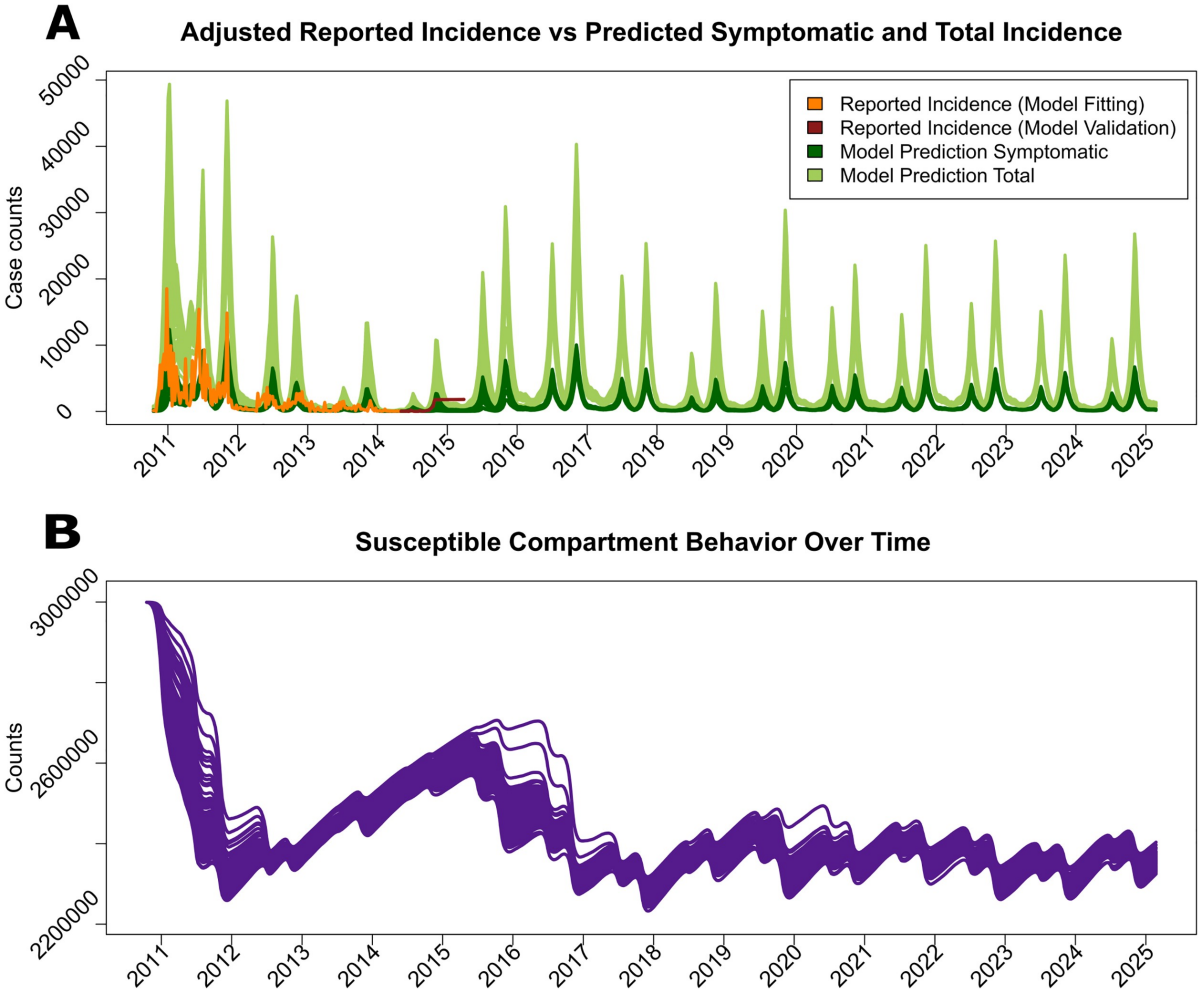
The cholera incidence simulated by the model is presented for the next 10 years without any intervention, along with the illustration of the number of susceptible population members. In the upper panel (A) the observed incidence of symptomatic cholera cases adjusted for under-reporting (orange), the model-fitted incidence of symptomatic cases (dark green), both symptomatic and asymptomatic cases (light green) are presented. Incidence data from October 2010 to April 2014 were used for estimation, and incidence data (dark red) from May 2014 until May 2015 were used to illustrate agreement with the model. The number of susceptible individuals (purple) is displayed in the lower panel (B).

In the absence of effective interventions, the initial outbreak grew in size and spread uncurbed throughout the entire country in epidemic form. Since the current data-driven dynamic model (4) estimates the concentration of toxigenic *V. cholerae* O1 in the environment using rainfall and temperature measurements, the extrapolation of future climatological data allowed for the simulation of future seasonal outbreaks in order to test different OCV interventions. The resulting number of future cholera cases and the punctuation of future epidemics during the rainy season are shown in Fig 3A. The number of new cases predicted by model (4) for the short period from May 4, 2014 until March 29, 2015, was in reasonable visual agreement with the adjusted reported incidence for the same time and created stable, synthetic epidemics (Fig 3A). Due to the temporary immunity from El Tor cholera infections, the number of susceptible humans in the Ouest Department eventually stabilizes at around two million people, leading to an adequate number of hosts to facilitate seasonal cholera epidemics into the distant future (Fig 3B).

### Effect of proactive vaccination campaigns on future epidemics

**Fig 4:**
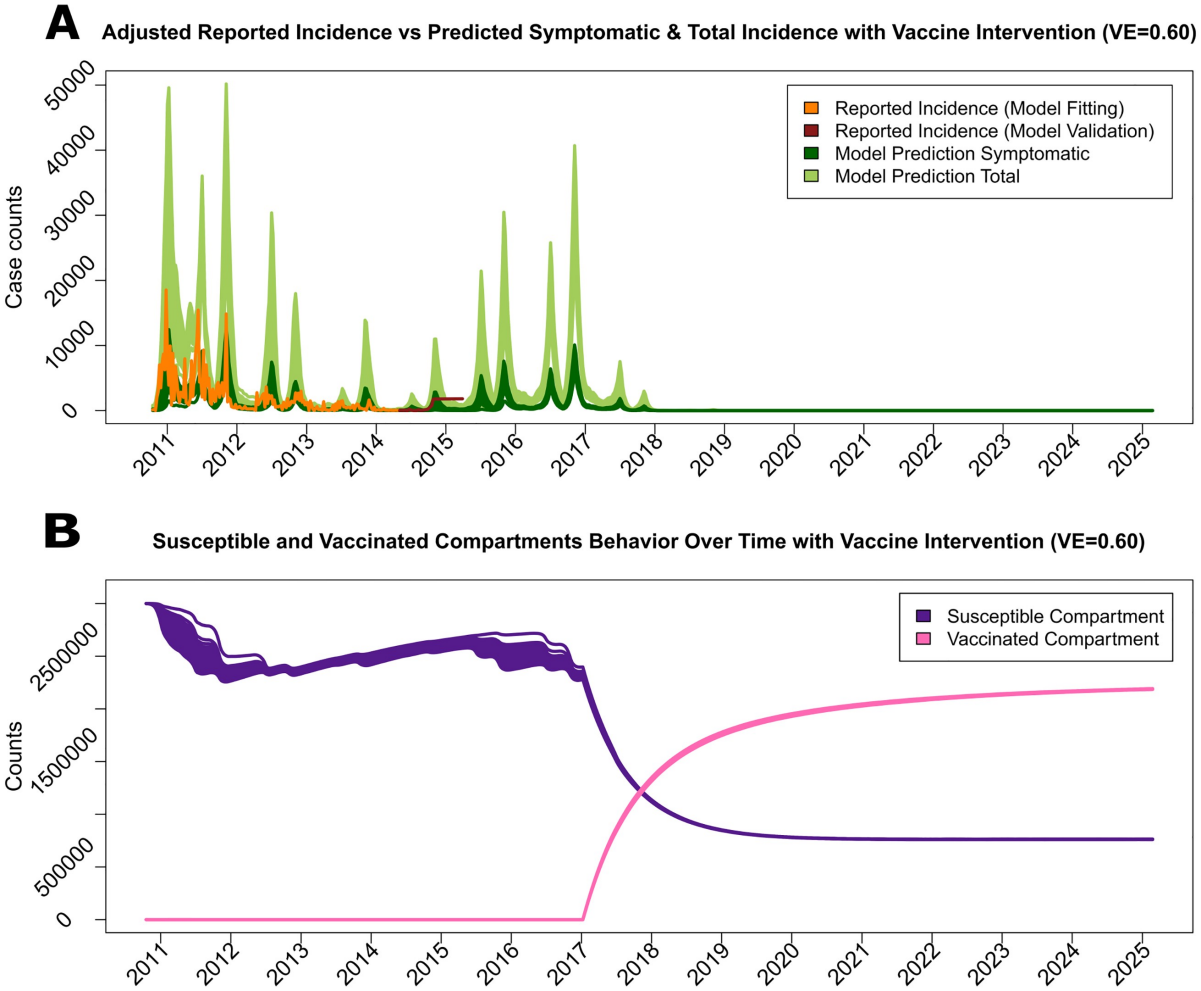
The cholera incidence simulated by the model is presented for the next 10 years with vaccine intervention, along with the number of susceptible and vaccinated population members. The intervention was initiated January 1, 2017, assuming a 60% vaccine efficacy, 3 year duration of immunity, and an average waiting time to be vaccinated of 50 weeks. The upper panel (A) presents the observed incidence of symptomatic cholera cases (orange and dark red), the model-fitted incidence of symptomatic cases (dark green), and both symptomatic and asymptomatic cases (light green). The lower panel (B) displays the numbers of susceptible (purple) and vaccinated (pink) individuals.

Multiple vaccination strategies were explored to verify their ability to control future cholera epidemics. The first scenario was a mass vaccination campaign set to commence on January 1, 2017, assuming each vaccination, when attempted with the rate *μ*_*SV*_, was successful with probability 1. In this campaign, the average waiting time for an individual to be vaccinated was 50 weeks, vaccine efficacy was set to 60%, and the duration of protective immunity lasted an average of three years. As shown in Fig 4A, cholera transmission was completely eliminated by May 20, 2018 (95% CI: January 7, 2018; September 30, 2018). Similar to the reactive vaccine campaign, this model suggested that the epidemic would be eliminated before all individuals were vaccinated due to reduction in transmission via herd immunity (Fig 4B). Two additional vaccination strategies were considered for sensitivity analysis. Both strategies are identical to the original setting except that 1.) the first additional strategy assumes that the probability of success for each attempted vaccination was 0.6, resembling non-perfect vaccine delivery due to any circumstances, and 2.) the second additional strategy delayed the campaign initiation to September 3, 2017. The reduction in the number of symptomatic cases for all three strategies are presented along with the number of susceptible and vaccinated individuals in Fig C and Fig D in S1 Text, respectively. The results from all three simulations indicated that even with lower probability of successful attempts or a later start date, any OCV with a relatively high efficacy (approximately 60%) could be used to successfully eliminate future cholera epidemics.

### Sensitivity analysis of proactive vaccination campaigns

**Fig 5:**
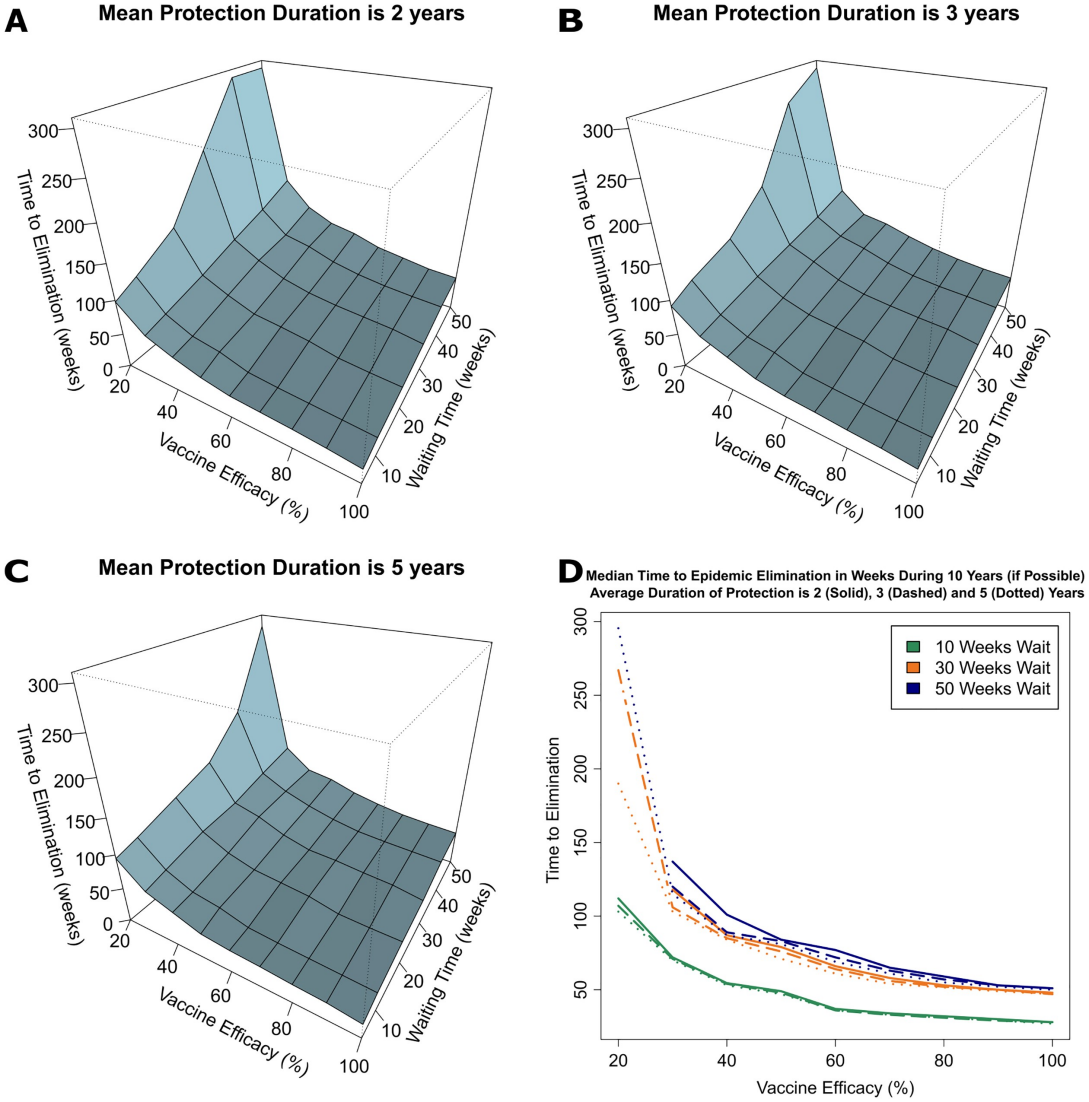
The median times to elimination of cholera transmission for parameter settings defined as combinations of vaccine efficacy (20-100% with 10% increments) and the average waiting time to be vaccinated (5, 10, 20, 30, 40 and 50 weeks) are presented, stratified by the average duration of immunity after vaccination of 2 (A), 3 (B), and 5 (C) years. Panel (D) shows slices of the three-dimensional plots at 10, 30, and 50 weeks for the average waiting time to be vaccinated. Times to elimination exceeding 300 weeks are truncated at 300 in panels (A)-(C) and not included in panel (D).

**Fig 6:**
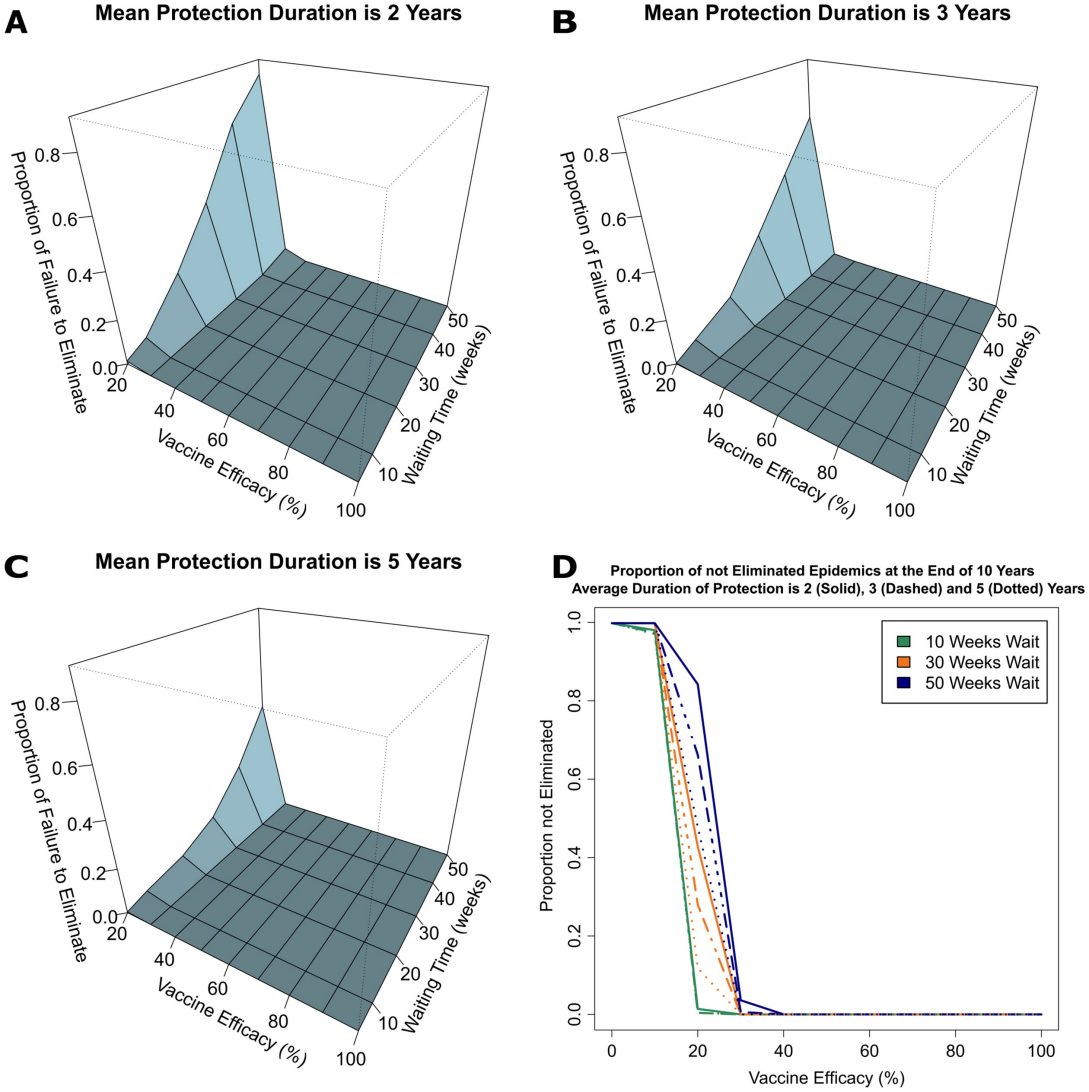
The proportion of failure to eliminate cholera transmission among all simulated epidemics for parameter settings defined as combinations of vaccine efficacy (20-100% with 10% increments) and the average waiting time to be vaccinated (5, 10, 20, 30, 40 and 50 weeks) are presented, stratified by the average duration of immunity after vaccination of 2 (A), 3 (B), and 5 (C) years. Panel (D) shows slices of the three-dimensional plots at 10, 30, and 50 weeks for the average waiting time to be vaccinated. Proportions of failure to eliminate higher than 0.8 are truncated at 0.8 in panels (A)-(C) but are shown in panel (D).

Sensitivity analyses were performed to evaluate the effects of a wide range of key parameters of proactive vaccination campaigns including vaccine efficacy (0% to 100%), average waiting time for an individual to be vaccinated (5 to 50 weeks), and duration of protective immunity (2, 3 or 5 years). Using these settings, the median times necessary to eliminate cholera transmission in the Ouest Department of Haiti (expressed in weeks from January 1, 2017, the onset of the campaign) are presented by the duration of protective immunity in Tables E, F and G in S1 Text and visualized in Fig 5. Additionally, the proportions of failure to eliminate transmission by 2023 with mass vaccination campaigns among simulated epidemics are provided in Fig 6. As expected, vaccination campaigns with lower vaccine efficacy or longer average waiting times for individuals to be vaccinated took longer to eliminate cholera transmission (Fig 5). Interestingly, only campaigns with the lowest levels of vaccine efficacy (≤30%) and longest vaccination waiting times (≥50 weeks) failed to eliminate cholera transmission by 2023. Likewise, the proportion of vaccine campaigns that failed to control cholera transmission were not significantly different when the vaccine efficacy was 30% or higher regardless of the waiting time to vaccination, provided the duration of protective immunity from the vaccine lasts at least three years (Figs 5 and 6). Using the reported vaccine efficacy of OCVs (60%) and the average duration of protective immunity of at least 3 years, campaigns with a maximum average waiting time for individuals to be vaccinated of 50 weeks, elimination of transmission occurred in all of the simulated epidemics with a median time of 22 to 72 weeks (Tables F and G in S1 Text). The corresponding cumulative vaccination coverages at different time points for this scenario is obtained from the first row of Table C where a 50-week waiting time is used. For example, 25% of the population has been vaccinated within 14.38 weeks and 50% of the population has been vaccinated by 34.66 weeks. The coverage at 22 weeks is interpolated to be about 35%.

## Discussion

In agreement with other vaccine intervention models of the great Haitian cholera epidemic [10] [27], the current study indicated that the use of OCVs in Haiti at the onset of the outbreak could have greatly reduced the morbidity and mortality from cholera and possibly curtailed the spread throughout Haiti. At that time, the use of OCVs had yet to be recommended as an appropriate method for the control of non-endemic cholera [18] and insufficient quantities were available from the manufacturers, making the implementation of any large-scale vaccinations impossible even if administration was restricted to only high-risk groups [17]. Despite the planned investment of almost half a billion dollars to improve drinking water and sanitation infrastructure in Haiti between 2013 and 2015 [28], there is little empirical evidence that any nationally coordinated efforts have been implemented since the earthquake [29]. Additionally, establishment of such systems would require long-term commitments, ongoing monitoring and maintenance, which compared to the relatively rapid protection conferred by OCVs, makes mass vaccination campaigns an attractive component of any plans for controlling cholera in Haiti.

Nevertheless, practical aspects of the implementation of a mass vaccination campaign in Haiti represent a formidable challenge. From Table C in S1 Text it follows that for an average waiting time of 30 weeks, 1.5 million people (50% of the population) are expected to be vaccinated within 20.79 weeks and 2.25 million people (75% of the population in the Ouest Department) within 41.59 weeks. This is also not taking into account that both vaccines currently provided by the strategic stockpile (Euvichol, Eubiologics Co. and Shancol, Shantha Biotechnics) have international protocols that requires two doses given at least two weeks apart [30]. Even though the addition of Euvichol OCV to the stockpile could increase the annual supply to approximately 10 million doses [31], there are over a billion people living in areas at risk for cholera [32]. Thus, it is unlikely the entire strategic stockpile would be allocated to a single country for the purposes of cholera elimination. Indeed, the limited availability of OCVs and the complex logistics of the administration of multi-dose regimen of vaccine that must be transported and maintained by a cold-chain were some of the main reasons that OCVs were not utilized during the initial outbreak [33]. However, a single dose of OCV has recently been demonstrated to be highly effective in the prevention of cholera transmission and the promotion of herd immunity in the population [34]. By targeting campaigns to high-risk populations, such as rural areas with high rates of surface water consumption that are difficult to reach for medical intervention [35], the use of fewer vaccines would likely be more effective than the mass administration of OCV simulated by the current model.

In reality, cholera vaccination pilot programs in Haiti began in early 2012, without the use of the strategic stockpile. Between April and June 2012, 200,000 doses of Shancol were purchased through funds obtained from the American Red Cross, after which 100,000 doses of OCV were administered by The Haitian Group for the Study of Kaposi’s Sarcoma and Opportunistic Infections (GHESKIO) in the urban slums of Port-au-Prince and Zanmi Lasante/Partners in Health (ZL/PIH) in the rural Artibonite Valley. In both demonstration projects, 99.9% of OCVs were administered successfully, over 90% of participants received both doses of OCV, an estimated vaccine coverage of 75% in at-risk communities was achieved, and the vaccine was well-tolerated with minimal side effects (less than 1%) [36] [37]. Not only was the OCV determined to be approximately 63% effective [38], the campaign was also associated with significant improvements in the knowledge of cholera transmission and the practice of cholera prevention methods in rural population; demonstrating that OCVs can encourage other cholera control efforts instead of hindering their progress [39]. More recently, the WHO has responded to the concerns of increased transmission of cholera after hurricane Matthew in October 2016 by allocating one million doses of Euvichol OCV to Haiti [41], though it has yet to ascertain the best method of administering these vaccines in the context of a ‘National Cholera Vaccination Program (NCVP)’.

Though this study was able to provide valuable insights on the impact that future vaccination campaigns could have on reducing cholera transmission in Haiti, it is important to mention some basic limitations that arise from the model assumptions. It was assumed that only susceptible individuals (*S*) would be vaccinated during the intervention, whereas the immunity of individuals (*S* or *R*) is often unknown and people who acquired immunity from previous infection would also be vaccinated. This would theoretically lead to the same result, but utilize more OCVs than necessary due to the redundant vaccination of individuals who acquired immunity through natural infection. The models assumed homogenous mixing within the population and equal risk of infection, whereas the differences in population density and access to improved water and sanitation throughout the Ouest Department are not evenly distributed. For simplicity, all vaccination strategies assumed that the average vaccine efficacy is the same for all individuals in the population, which is likely not the case. Similarly, the inclusion of anomalous rainfall events such as hurricanes (ie. Matthew in October 2016) were not included, hence the use of smoothed precipitation estimates could have affected the size of future cholera outbreaks and the time to elimination under vaccine interventions. The model also assumed that no improvements to drinking water and sanitation infrastructure will be implemented, and that the growth patterns of the lytic phages and toxigenic *V. cholerae* O1 in the environment will remain stable. These assumptions, though reasonable, could change in the future due to the documentation of the shift from Ogawa to Inaba serotype of *V. cholerae* O1 identified in the environment and human samples collected from the Ouest Department [40]. If such a phase-change has happened, then the antibodies formed to one serotype offer less protection against infections from a different serotype and another large scale epidemic could follow in the near future. This would provide an even stronger rationale for proactive vaccination campaigns to commence as soon as possible, as the bi-valent OCV confers protection from both Ogawa and Inaba serotypes. Lastly, in this study we only considered the Ouest Department because of the availability of matched environmental surveillance and incidence data used to explore the underlying transmission dynamics. It must be understood that, for national control of cholera using mass administration of OCVs to be successful, campaigns would have to be initiated in all ten departments to prevent re-introduction after the duration of protective immunity post-vaccination has waned.

## Conclusion

Improvements in drinking water and sanitation infrastructure should remain a priority for future development due to their long-term effectiveness at preventing cholera and other water-borne diseases. In the meantime, OVCs could be administered in mass vaccination campaigns or to targeted high risk-groups with or without the use of the strategic stockpile at a relatively low cost (approximately $2 USD/dose). Though the future of the Haitian cholera epidemic remains uncertain, the results from this study indicate that even with some variability in vaccine efficacy and rates of OCV administration, elimination of cholera transmission in the Ouest Department of Haiti could be possible in a timeframe of only a few years.

## Supporting information

S1 Text Model details. Additional information about the data used, model fitting and outputs.
(PDF)
