# Supplementary material for: Controlling cholera in the Ouest Department of Haiti using oral vaccines
Source: PLoS Negl Trop Dis. 2017 Apr 14;11(4):e0005482. doi: 10.1371/journal.pntd.0005482 (PMC5406029; doi:10.1371/journal.pntd.0005482)
Supplement: S1 Text — Additional information about the data used, model fitting and outputs. (PDF) [file pntd.0005482.s001.pdf]

S1 Text: Model Details

Parameters Used in the Model

To ensure identifiability of the model selected parameters were assigned fixed values based on biological processes obtained via literature review [1] [2] [3] [4] [5]. The summary of the parameters is presented in Table A. The population size N considered for the simulation was 3 million people, which was based on a Central Intelligence Agency (CIA) estimates of the population of Ouest Department at that time of the initial outbreak in 2010.

Table A. Parameters fixed when fitting the model.

Parameter	μ_{AR}	μ_{IR}	μ_{AW}	$\mu_{IW} = 100\mu_{AW}$	κ	χ
Value	1	1	0.07	7	10^5	10^6

Simulation Settings for the Epidemics

Simulations were written in `C++` programming language. Direct Gillespie algorithm was used for simulations with the parameters obtained during the LSE approach [20][26]. The set of parameters obtained from the deterministic LSE solution of differential equations [20] was used for the simulations since the model produced the most stable results for prediction of long-term epidemics.

The set of the parameters used for the simulations is presented in Table B. The starting points for the compartments S, A, I, R, W were generated from the uniform distribution with the mean equal to the actual number of cases at the beginning of the epidemic and the minimum and maximum values equal 0 and two times the mean, respectively. The environmental compartment W was updated deterministically on every Gillespie step with the change equal to the rate of the exponential distribution multiplied by the transition time. The sign of the change indicated growth or death of bacteria, respectively.

Table B. Parameters used for epidemic simulations.

Estimated Parameter	Estimated Value
$\log(\mu_{SI}^{(W)})$	-4.349
$\log(\mu_{SA}^{(W)})^{\dagger}$	-3.250
$\log(\mu_{SI}^{(H)})$	-25.001
$\log(\mu_{SA}^{(H)})^{\ddagger}$	-23.901
$\log(\mu_{RS})$	-5.114
$\log(\mu_{W-})$	0.941
$\log(\alpha)$	-19.999
β	0.020
$\log(\rho_c)$	3.807
$\log(\sigma)$	2.707
$\log(\delta)$	3.701

$\dagger$ $\log(\mu_{SA}^{(W)}) = \log(3\mu_{SI}^{(W)})$

$\ddagger$ $\log(\mu_{SA}^{(H)}) = \log(3\mu_{SI}^{(H)})$

Alternative Vaccination Strategies

To investigate how the choice of the starting date would affect the efficacy of the vaccination campaign, two alternative vaccination strategies were considered. The first alternative used the same starting date for the vaccination (January 1, 2017), but the probability of being vaccinated was made equal to 0.6. The second strategy used September 3, 2017 as the starting date, since it was right before the predicted epidemic spike.

Environmental Data Used for the Future Dates

The dependence of the epidemic on the values of the covariates (temperature and precipitation) allowed us to predict the future behavior of the epidemic, however the values of temperature and precipitation were obviously unknown for the future times. Since the daily temperature and precipitation were available for almost four years, three to four readings for each calendar day were available for extrapolation of future values. To fill future dates, the average value of the corresponding temperature and precipitation reading for each day obtained during the observation period years was used. The only exception was February 29, 2012 since it occurred during a leap year and only had a single value. The averaged time series from June 22, 2014 until June 20, 2025 were produced and are presented in Supplementary Fig. B and Supplementary Fig. A. The daily readings for the future data were aggregated to weekly measurements in the same fashion as for observed data i.e. average temperature in degrees Celsius and total weekly precipitation in millimeters.

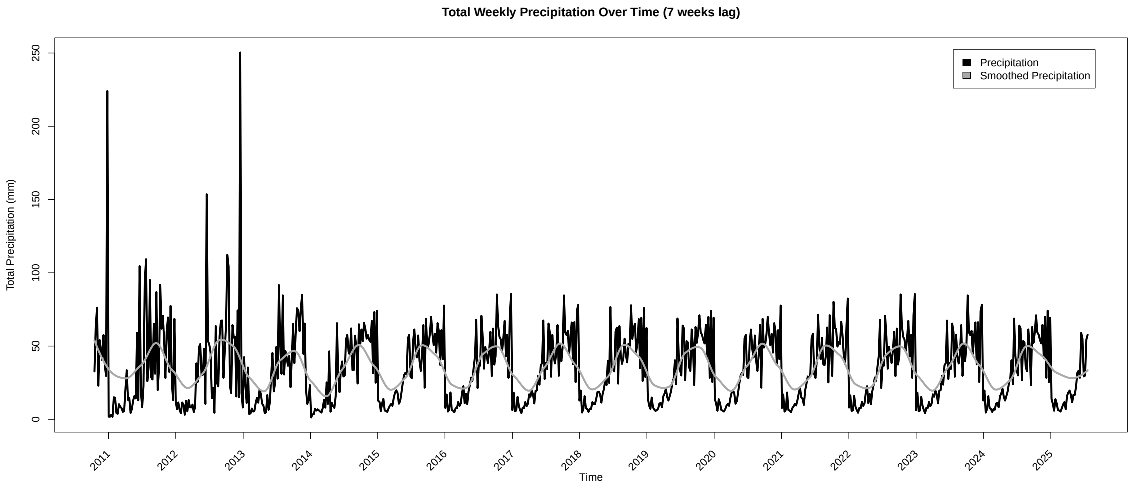

Figure A. The observed and extrapolated future precipitation readings (7 weeks lag) until 2025 are presented (black) with a polynomial smoother (gray).

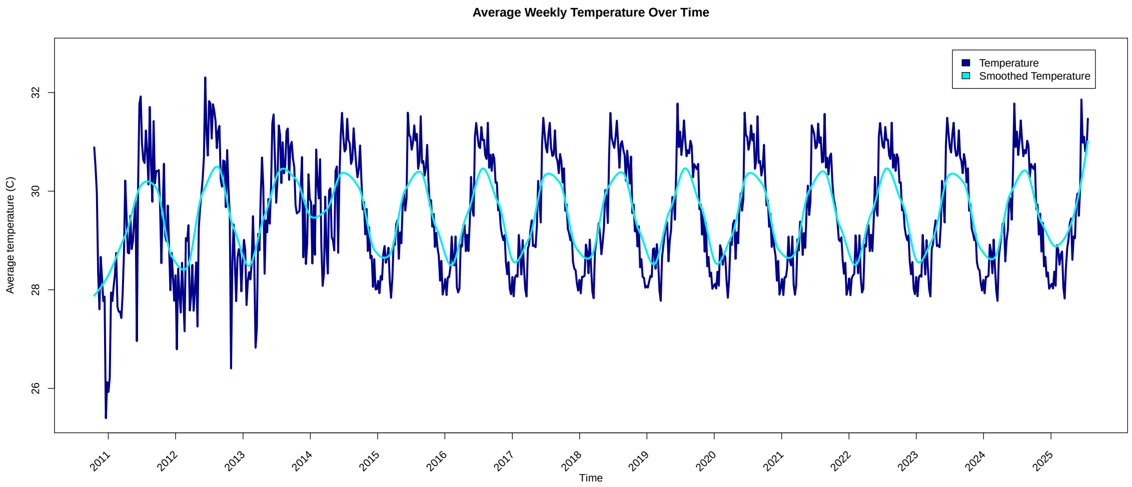

Figure B. The observed and extrapolated future temperature readings until 2025 are presented (blue) with a polynomial smoother (cyan).

Table C. The quantiles of the exponential distribution in weeks for the given average waiting time for the vaccine. The number of weeks corresponds to the time by which the given proportion of population is expected to be vaccinated.

Expected Wait	Percentage of population					
(Weeks)	0%	25%	50%	75%	95%	99%
50	0	14.38	34.66	69.31	149.79	230.26
40	0	11.51	27.72	55.45	119.83	184.21
30	0	8.63	20.79	41.59	89.87	138.16
20	0	5.75	13.86	27.73	59.91	92.10
10	0	2.88	6.93	13.86	29.96	46.05
5	0	1.44	3.47	6.93	14.98	23.02

Table D. The quantiles of the exponential distribution in years for the given average duration of protection induced by the vaccine. The number of years in the table corresponds to the proportion of people expected to lose the immunity by the given time.

Duration of Protection	Percentage of study population					
(Years)	0%	25%	50%	75%	95%	99%
2.0	0	0.58	1.39	2.77	5.99	9.21
3.0	0	0.86	2.08	4.16	8.99	13.82
5.0	0	1.44	3.47	6.93	14.99	23.02

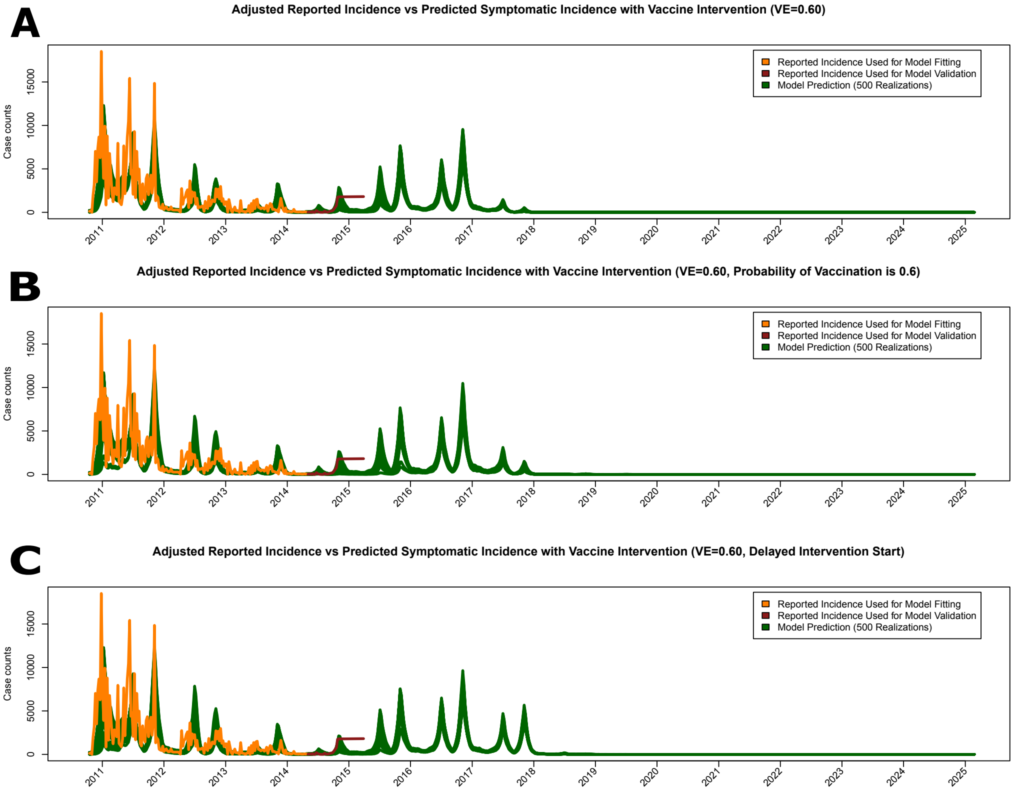

Figure C. The simulation results of three alternative vaccine intervention strategies are presented. In panels (A)-(C) the observed incidence of symptomatic cholera cases adjusted for under-reporting (orange) and the model-fitted incidence of symptomatic cases (dark green), both symptomatic and asymptomatic cases (light green) are presented. Incidence data from October 2010 to April 2014 was used for estimation, with incidence data (dark red) from May 2014 until May 2015 used for model validation. Panel (A) uses the simulation settings presented in Figure 4; compared to panel (A), panel (B) uses a different probability of being vaccinated (0.6 versus 1); compared to panel (A), panel (C) uses a start date of September 3, 2017 instead of January 1, 2017. The figures display 500 simulated epidemics produced by the model.

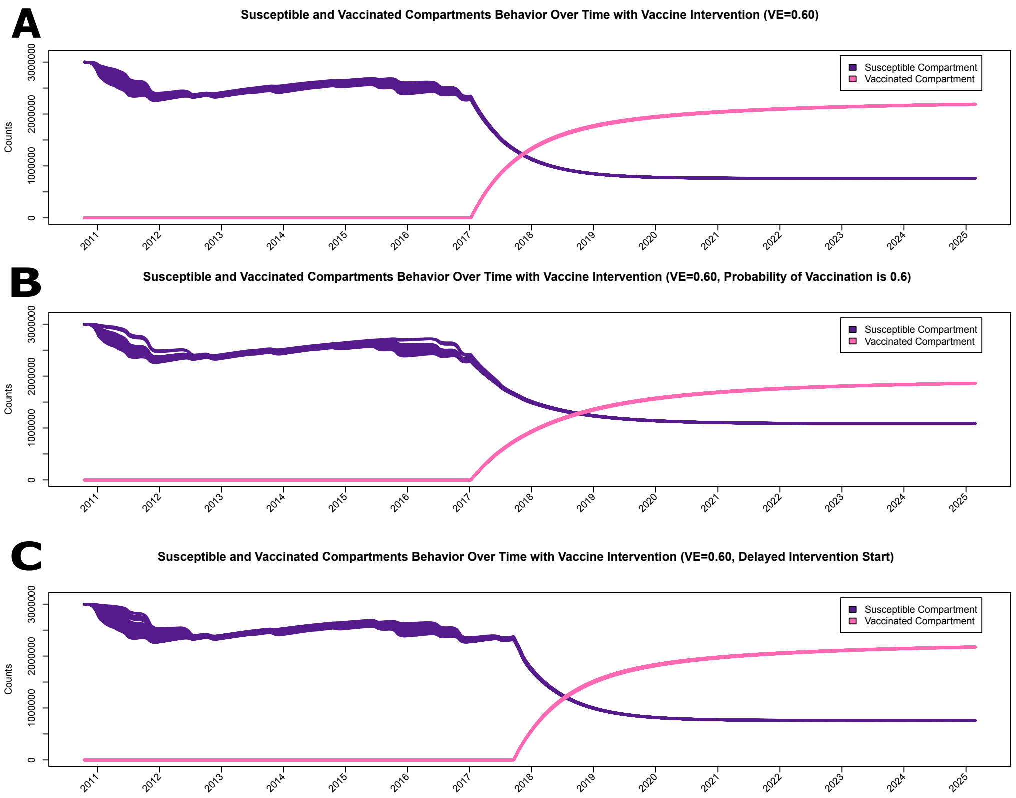

Figure D. The number of susceptible (purple) and vaccinated (pink) individuals during three alternative vaccine intervention strategies are presented. Panel (A) uses the simulation settings presented in Figure 4; compared to panel (A), panel (B) uses a different probability of being vaccinated (0.6 versus 1); compared to panel (A), panel (C) uses a start date of September 3, 2017 instead of January 1, 2017. The figures display 500 simulated epidemics produced by the model.

Table E. The median estimated time to eliminate cholera epidemics is presented with confidence intervals (2.5th and 97.5th percentiles computed from 500 simulations) using an average duration of vaccine-induced immunity equal to 2 years. NA corresponds to simulations that fail to eliminate the epidemics by 2025. Simulations that fail to eliminate the epidemic by 2025 were included in the median time computation.

Vaccine Efficacy	Expected waiting time for the vaccine (weeks)					
(VE) in %	5	10	20	30	40	50
0*	NA (NA;NA)	NA (NA;NA)	NA (NA;NA)	NA (NA;NA)	NA (NA;NA)	NA (NA;NA)
0.1	NA (NA;NA)	NA (NA;NA)	NA (NA;NA)	NA (NA;NA)	NA (NA;NA)	NA (NA;NA)
0.2	98 (61; 231)	112 (76;NA)	144 (85;NA)	223 (86;NA)	NA (104;NA)	NA (137;NA)
0.3	65 (48; 107)	72 (52; 106)	86 (60; 151)	103 (78; 176)	118 (82; 222)	137 (85;NA)
0.4	51 (33; 67)	55 (44; 82)	69 (51; 93)	82 (56; 113)	87 (63; 138)	101 (75; 157)
0.5	40 (30; 56)	49 (33; 60)	55 (45; 80)	66 (51; 87)	79 (56; 104)	84 (61; 118)
0.6	33 (29; 48)	37 (31; 51)	51 (34; 66)	57 (48; 82)	66 (51; 86)	77 (54; 101)
0.7	31 (26; 37)	34 (29; 47)	46 (32; 54)	52 (41; 68)	58 (48; 80)	65 (51; 84)
0.8	29 (22; 34)	32 (28; 39)	38 (31; 50)	49 (34; 59)	53 (45; 71)	59 (49; 80)
0.9	26 (18; 31)	30 (25; 34)	35 (30; 48)	46 (33; 54)	50 (36; 64)	53 (47; 73)
1**	22 (15; 28)	28 (23; 32)	33 (29; 44)	40 (32; 51)	48 (34; 56)	51 (44; 65)

* The efficacy 0 corresponds to no intervention.
** The efficacy 1 corresponds to the ideal vaccine.

Table F. The median estimated time to eliminate cholera epidemics is presented with confidence intervals (2.5th and 97.5th percentiles computed from 500 simulations) using an average duration of vaccine-induced immunity equal to 3 years. NA corresponds to simulations that fail to eliminate the epidemics by 2025. Simulations that fail to eliminate the epidemic by 2025 were included in the median time computation.

Vaccine Efficacy	Expected waiting time for the vaccine (weeks)					
(VE) in %	5	10	20	30	40	50
0*	NA (NA;NA)	NA (NA;NA)	NA (NA;NA)	NA (NA;NA)	NA (NA;NA)	NA (NA;NA)
0.1	NA (202;NA)	NA (NA;NA)	NA (NA;NA)	NA (NA;NA)	NA (NA;NA)	NA (NA;NA)
0.2	92 (62;226)	107 (69;NA)	128 (83;NA)	173 (91;NA)	267 (103;NA)	NA (NA;NA)
0.3	64 (48; 98)	71 (52; 106)	84 (59; 128)	95 (72; 157)	106 (81; 193)	120 (83; 243)
0.4	51 (33; 68)	54 (37; 79)	65 (51; 86)	80 (55; 109)	85 (62; 118)	89 (71; 137)
0.5	38 (31; 52)	48 (32; 62)	54 (46; 80)	64 (50; 85)	76 (53; 100)	83 (59; 109)
0.6	33 (28; 49)	36 (31; 52)	50 (34; 62)	55 (46; 74)	64 (51; 84)	72 (53; 91)
0.7	31 (26; 38)	33 (28; 46)	46 (32; 54)	51 (40; 65)	56 (48; 78)	63 (50; 84)
0.8	28 (22; 33)	31 (27; 38)	37 (31; 50)	48 (34; 59)	52 (44; 68)	57 (48; 78)
0.9	26 (18; 30)	29 (25; 34)	34 (30; 47)	46 (33; 52)	50 (34; 62)	53 (46; 70)
1**	22 (15; 27)	28 (22; 32)	33 (29; 44)	39 (31; 50)	47 (33; 53)	51 (40; 63)

* The efficacy 0 corresponds to no intervention.
** The efficacy 1 corresponds to the ideal vaccine.

Table G. The median estimated time to eliminate cholera epidemics is presented with confidence intervals (2.5th and 97.5th percentiles computed from 500 simulations) using an average duration of vaccine-induced immunity equal to 5 years. NA corresponds to simulations that fail to eliminate the epidemics by 2025. Simulations that fail to eliminate the epidemic by 2025 were included in the median time computation.

Vaccine Efficacy	Expected waiting time for the vaccine (weeks)					
(VE) in %	5	10	20	30	40	50
0*	NA (NA;NA)	NA (NA;NA)	NA (NA;NA)	NA (NA;NA)	NA (NA;NA)	NA (NA;NA)
0.1	NA (208;NA)	NA (NA;NA)	NA (NA;NA)	NA (NA;NA)	NA (NA;NA)	NA (NA;NA)
0.2	95 (61; 208)	103 (67;360)	122 (82;NA)	141 (86;NA)	190 (101;NA)	295 (106;NA)
0.3	63 (48; 89)	70 (51; 102)	83 (58; 119)	89 (71; 164)	103 (80; 164)	115 (83; 191)
0.4	50 (33; 66)	53 (37; 78)	64 (50; 86)	79 (54; 103)	84 (60; 119)	87 (68; 132)
0.5	37 (30; 52)	47 (32; 58)	53 (45; 74)	63 (50; 84)	71 (52; 91)	81 (58; 102)
0.6	33 (28; 49)	36 (30; 51)	49 (33; 62)	53 (45; 76)	61 (50; 83)	69 (52; 87)
0.7	31 (26; 35)	33 (29; 45)	45 (32; 52)	51 (35; 62)	54 (47; 76)	61 (50; 82)
0.8	28 (21; 32)	31 (27; 37)	36 (31; 48)	47 (33; 54)	51 (44; 64)	55 (47; 78)
0.9	26 (18; 30)	29 (25; 33)	34 (30; 46)	44 (32; 52)	49 (34; 56)	52 (45; 67)
1**	21 (15; 27)	27 (22; 31)	33 (28; 42)	37 (31; 49)	47 (33; 52)	50 (38; 60)

* The efficacy 0 corresponds to no intervention.
** The efficacy 1 corresponds to the ideal vaccine.

References

1. Codeco CT (2001) Endemic and epidemic dynamics of cholera: The role of the aquatic reservoir. BMJ Infect Dis 1:1
2. Righetto L, Bertuzzo E, Mari L, Schild E, Casagrandi R, Gatto M, Rodriguez-Iturbe I, Rinaldo A (2013) Rainfall mediations in the spreading of epidemic cholera. Advances in Water Resources.
3. Kaper JB, Morris JG Jr, Levine MM (1995) Cholera. Clin Microbiol Rev. Jan;8(1):48-86.
4. Nelson EJ, Harris JB, Calderwood SB, Camilli A (2009) Cholera transmission: the host, pathogen and bacteriophage dynamic. Nat Rev Microbiol 7:693-702.
5. Fung IH (2014) Cholera transmission dynamic models for public health practitioners. Emerging Themes in Epidemiology 11(1):1-11.
